# Measurements of Radical Reactivity with an Imine, (CF_3_)_2_CNH: Rate Constants for Chlorine Atoms and Hydroxyl Radicals and the Global Warming Potential

**DOI:** 10.3390/molecules31030424

**Published:** 2026-01-26

**Authors:** Savi Savi, Paul Marshall

**Affiliations:** Department of Chemistry and Center for Advanced Scientific Computing and Modeling, University of North Texas, 1155 Union Circle #305070, Denton, TX 76203, USA

**Keywords:** imine, rate constant, relative rate, climate metric

## Abstract

The rate constant *k*_OH_ for the reaction of 1,1,1,3,3,3-hexafluoroprop-2-imine with OH radicals was measured relative to two reference compounds, CH_3_F and CH_3_CHF_2_, to be *k*_OH_ = (4.2 ± 1.1) × 10^−14^ cm^3^ molecule^−1^ s^−1^ at 295 K. This implies an atmospheric lifetime with respect to consumption by OH of 0.75 years. Reaction with Cl atoms yielded *k*_Cl_ = (7.9 ± 1.7) × 10^−16^ cm^3^ molecule^−1^ s^−1^ at 295 K, and reaction with O_3_ has an upper limit of *k*_O3_ < 4 × 10^−23^ cm^3^ molecule^−1^ s^−1^, so that the atmospheric consumption by Cl and O_3_ is negligibly slow. Absolute infrared cross sections of the imine yield a radiative efficiency of 0.34 W m^−2^ ppb^−1^, which is corrected to 0.23 W m^−2^ ppb^−1^ for the effects of atmospheric lifetime. The imine’s corresponding 100-year global warming potential is 64 ± 19. This value is an upper limit, given that heterogenous atmospheric removal paths, such as hydrolysis in water droplets, are not included.

## 1. Introduction

Efforts to reduce the carbon intensity of materials used industrially, and, more generally, to contribute to a low-carbon society, require evaluation of climate metrics for emitted compounds. This information can guide decisions and regulations that affect the environment, notably the Montreal Protocol [1]. The earth’s temperature is controlled by a balance between the absorption of incoming solar radiation by the earth, primarily in the visible region of the spectrum, and outgoing radiation of energy from the earth’s surface in the infrared (IR) region. This latter IR emission is increasingly absorbed by growing atmospheric concentrations of greenhouse gases, including carbon dioxide, methane, nitrous oxide, and halogenated hydrocarbons. An approximate measure of the impact of a compound is its global warming potential (GWP). This metric reflects a combination of the ability of molecules to absorb infrared (their radiative efficiency) and their lifetime in the atmosphere (controlled by chemical and physical processes that vary with each molecule), evaluated relative to CO_2_ [2].

Saturated fluorinated molecules have been widely used as solvents, refrigerants, and sources of fluorine atoms in the plasma etching steps of microelectronics manufacture, but have high GWPs. Now, popular substitutes in some applications are hydrofluoroolefins, where the C=C π bond provides a site for rapid attack by atmospheric radicals, especially hydroxyl (OH), which shortens the lifetime in the environment and hence the GWP. Imines, which contain an analogous C=NH group, might offer an alternative class of low-GWP compounds and there has been interest in their application as plasma etchants [3]. However, there is essentially no information available about their atmospheric chemistry.

This report describes measurements that yield the GWP for (CF_3_)_2_CNH, formally named 1,1,1,3,3,3-hexafluoroprop-2-imine (denoted as HFPI here) or hexafluoroacetone imine. The work consists of determinations of (a) the IR spectrum of HFPI, (b) its reactivity with the dominant atmospheric oxidant, hydroxyl (OH) radicals, (c) any contribution to HFPI atmospheric consumption by reaction with a second radical, atomic chlorine, (d) the products of radical attack on HFPI, and (e) its lifetime in the atmosphere, including possible loss by photolysis, reaction with ozone and rainout, and the associated GWP.

More broadly, kinetic and mechanistic information about imines is also relevant to the high-temperature chemistry of nitrogen-containing compounds, where the species HNNH and CH_2_NH have been studied computationally [4,5,6,7,8,9], and the results have been incorporated into multireaction models to analyze, for example, the combustion of amines and especially ammonia, which has attracted much attention as a possible carbon-free fuel [10,11]. There is also a fundamental motivation for the experiments, since HFPI is the first example of an imine of the form R=N-H whose reactivity with radicals has been measured.

## 2. Results

### 2.1. Infrared Spectrum

**Figure 1:**
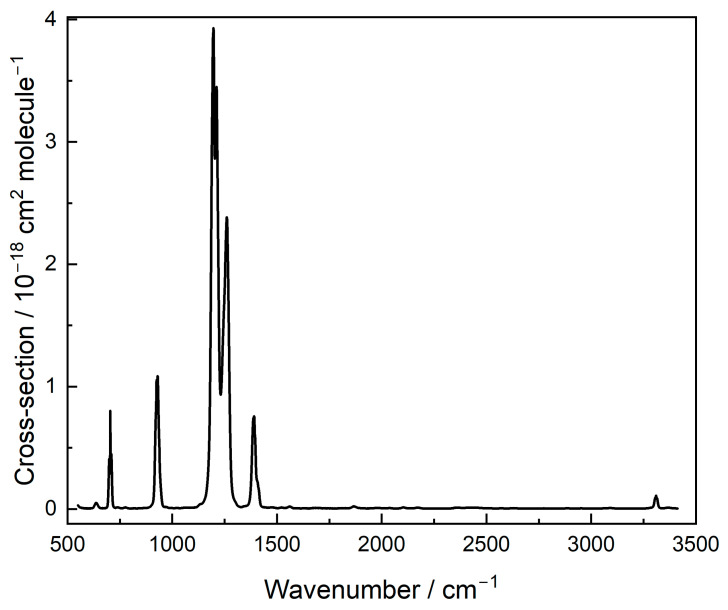
IR spectrum for HFPI.

Spectra of HFPI were recorded at five concentrations. Data for each wavenumber point in the scan were fit to the Beer–Lambert Law (see Section 4.2) to obtain the cross sections shown in Figure 1, which are tabulated in the Supplementary Materials.

These are the first absolute IR intensities available, and the peak positions agree with published values [12]. There are no experimental bands unaccounted for, suggesting good purity of the sample.

**Figure 2:**
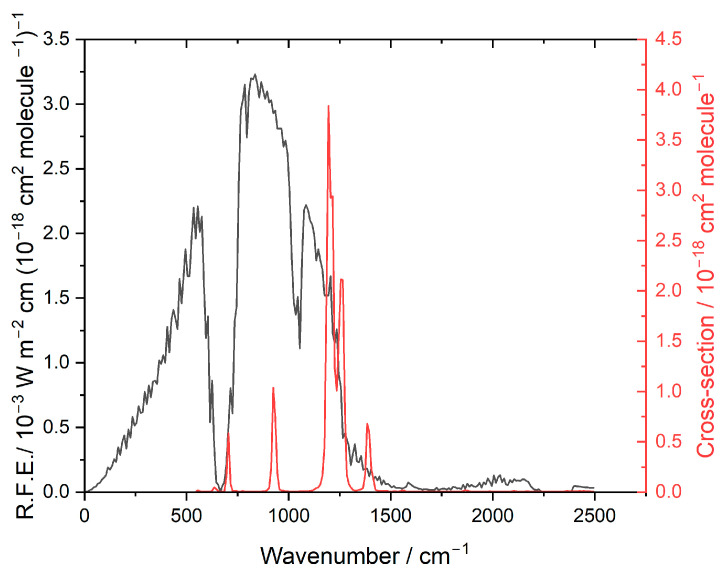
Earth’s radiation (RFE) plotted in black, left-hand scale. Absorption cross section of HFPI plotted in red, right-hand scale.

Figure 2 shows the earth’s outgoing IR radiation, which very roughly corresponds to blackbody emissions from an average surface temperature of ~290 K. The large dip near 700 cm^−1^ is the absorption of outgoing IR radiation by carbon dioxide (CO_2_), and the dip near 1000 cm^−1^ is caused by absorption by ozone (O_3_).

The overlap between this emission and the absorption by HFPI defines the radiative efficiency (RE), which is evaluated as 0.34 W m^−2^ ppb^−1^ [2], before a lifetime correction (see Section 3.2). The spectrum computed as described in Section 4.3 revealed that there are two fundamental frequencies below the instrumental cutoff of 550 cm^−1^ (at 479 and 532 cm^−1^), which could in principle affect the RE calculations [13]. We used calculated band strengths and found that their contribution to the overall RE is negligible, about 1%.

### 2.2. Reactivity of HFPI with Ozone and Hydroxyl Radicals

Hydroxyl radicals were created via UV photolysis of O_3_ at 254 nm. This forms electronically excited O(^1^D) atoms, which reacted with added hydrogen to make OH and a H atom. These H atoms in turn reacted with O_3_ to make further OH:

O_3_ + hν → O_2_ + O(^1^D)

O(^1^D) + H_2_ → H + OH

H + O_3_ → O_2_ + OH

**Figure 3:**
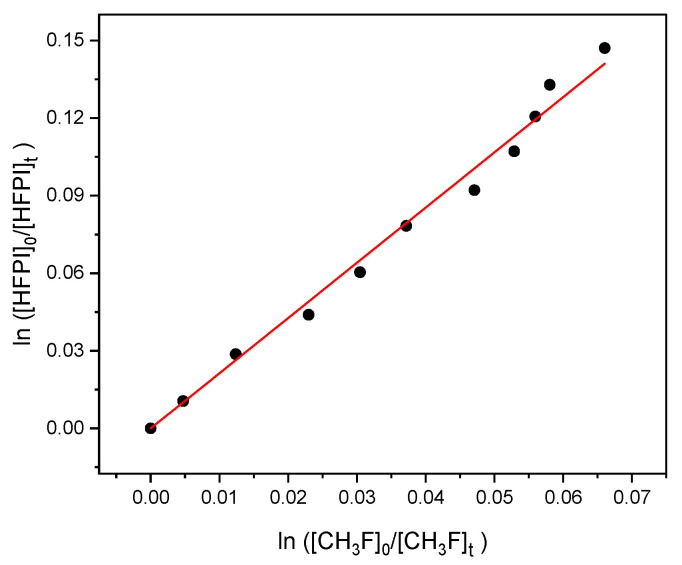
Relative consumption of HFPI and CH_3_F by OH radicals. Each point results from 38 co-added IR scans collected for 1 min, at intervals of 3 min.

Excess H_2_ ensured that O(^1^D) reacts with that and not the organic species. There is significant energy release and vibrationally excited OH molecules are formed initially; then, they are quenched to the ground state through collisions with the bath gas more quickly than they reacted with the added organic compounds. Relative rate experiments (see Section 4.2) were carried out at 294 ± 2 K to derive the OH kinetics with HFPI using two different reference species. One was fluoromethane (CH_3_F), monitored by its band at 2932–2954 cm^−1^. HFPI was monitored via its 1380–1400 cm^−1^ band. The total pressure of 1 bar was made up with N_2_ bath gas. Before UV light was applied to the reaction mixture, it was kept in the dark for 2.5 h. Over this time, [O_3_] dropped by 36% to a final value of ~8 × 10^16^ molecule cm^−3^, while [HFPI] dropped by 3%. This sets an upper limit to the bimolecular rate constant *k*_O3_ for reaction of O_3_ with HFPI as *k*_O3_ < 4 × 10^−23^ molecule^−1^ cm^3^ s^−1^. With the lamp turned on, OH began to consume HFPI and the reference, and IR spectra tracked the decrease in their absorbances at intervals of 3 min. A typical plot of relative consumption is shown in Figure 3, whose slope is *k*_HFPI_/*k*_ref_ (see Equation (2) in Section 4.2). Three experiments like this are summarized in Table 1.

The mean ratio is 2.17 with 0.07 or 3.4% for twice the standard deviation, 2σ. At 295 K, *k*_CH3F + OH_ is recommended as (1.91 ± 0.40) × 10^−14^ molecule^−1^ cm^3^ s^−1^, with 21% uncertainty (2σ) [14]. Combining these uncertainties in quadrature along with an allowance of 5% for potential instrumental uncertainty leads to a derived *k*_HFPI + OH_ at 294 K of (4.15 ± 0.91) × 10^−14^ molecule^−1^ cm^3^ s^−1^. The uncertainties provided here are 2σ.

**Figure 4:**
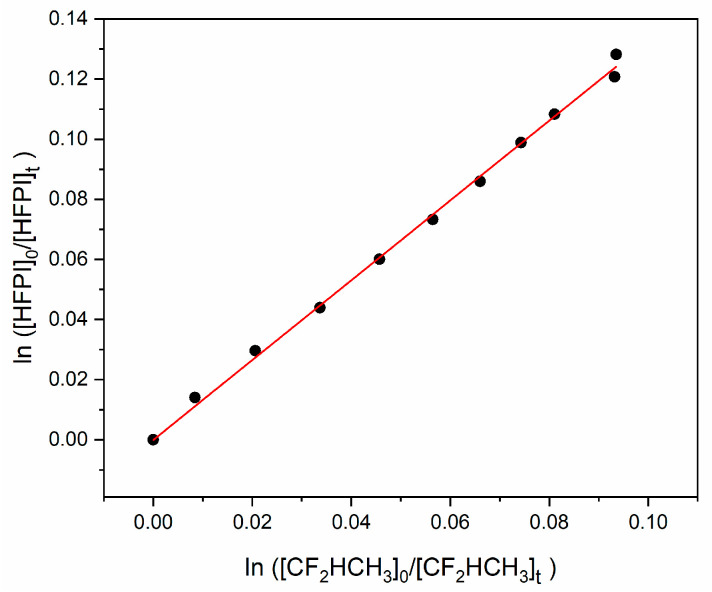
Relative consumption of HFPI and CH_3_CHF_2_ by OH radicals.

Similarly, 1,1 difluoroethane, CH_3_CHF_2_, was used as a second reference monitored at 2966–2984 cm^−1^, with HFPI monitored via its 698–708 cm^−1^ band, and the results are summarized in Table 2. A relative rate plot of HFPI consumption is shown in Figure 4.

The mean ratio is 1.35 with twice the s.d. equal to 0.03 or 2.2%. At 294 K, *k*_CH3CHF2+OH_ is recommended as (3.16 ± 0.46) × 10^−14^ molecule^−1^ cm^3^ s^−1^ with 15% uncertainty [14], so the derived *k*_HFPI+OH_ at 294 K is (4.27 ± 0.67) × 10^−14^ molecule^−1^ cm^3^ s^−1^. The two sets of experiments with different reference compounds agree within the uncertainties. From the weighted mean of the two results, we assess *k*_HFPI+OH_ = (4.21 ± 1.13) × 10^−14^ cm^3^ molecule^−1^ s^−1^ where all uncertainties are ±2σ.

### 2.3. Reactivity of HFPI with Chlorine Atoms

Molecular chlorine was employed as the source of Cl atoms, generated by photolysis at 365 nm. The reference compound 1,1,1,2-tetrafluoroethane was used, in a similar way as in the OH studies. CF_3_CH_2_F was monitored via its 1286–1304 cm^−1^ band and the reactant HFPI was monitored in the 1380–1400 cm^−1^ region. Initial checks showed negligible reaction in the absence of UV light.

**Figure 5:**
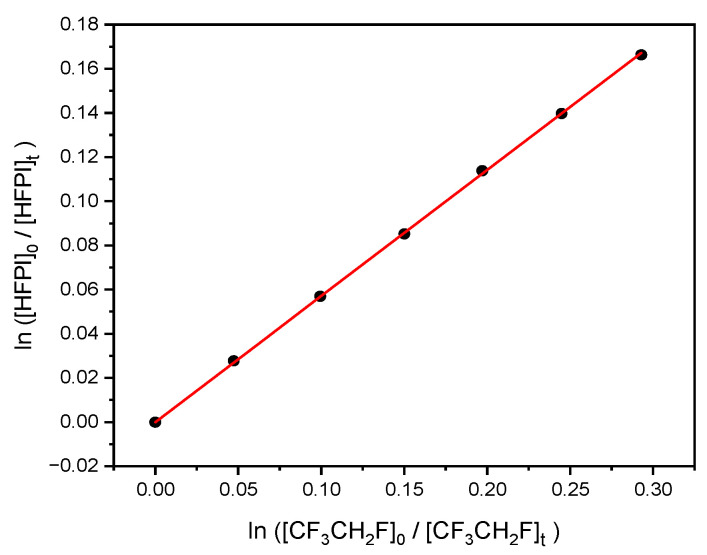
Relative consumption of HFPI and CF_3_CH_2_F by Cl radicals.

Experiments were conducted at 295 ± 2 K and 1 bar pressure balanced by N_2_ bath gas. A total of 38 IR scans were co-added for each spectrum that was collected for about one minute. The delay between collecting two consecutive spectra was 9 min. An example is shown in Figure 5 and three experiments are summarized in Table 3.

The mean ratio is 0.57 with 0.01 or 1.8% for twice the s.d. At 295 K, *k*_CF3CH2F_ is recommended as (1.39 ± 0.29) × 10^−15^ cm^3^ molecule^−1^ s^−1^, with 21% uncertainty at 2σ [14]. Combining these uncertainties in quadrature leads to a derived *k*_HFPI+Cl_ = (7.92 ± 1.71) × 10^−16^ cm^3^ molecule^−1^ s^−1^.

### 2.4. Products of HFPI Oxidation

**Figure 6:**
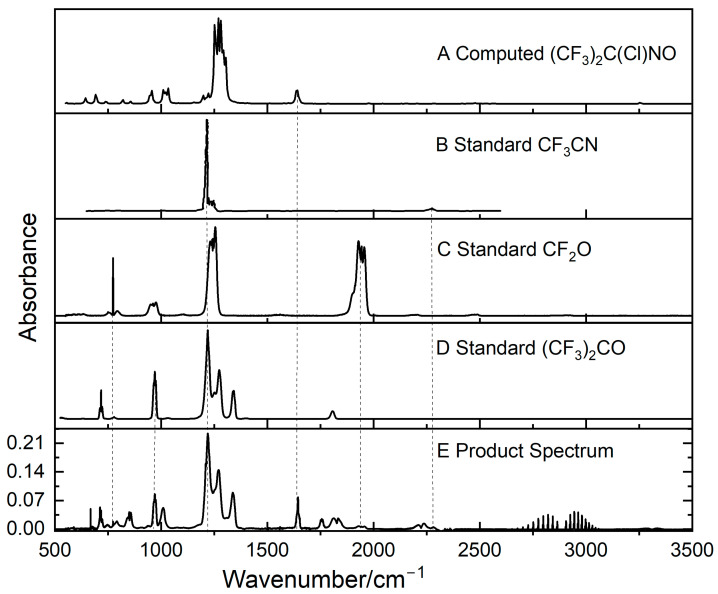
(**A**) Computed spectrum for (CF_3_)_2_C(Cl)NO, (**B**) reference spectrum for CF_3_CN [15], (**C**) reference spectrum for CF_2_O, (**D**) reference spectrum of (CF_3_)_2_CO [16], and (**E**) product spectrum after irradiating HFPI/Cl_2_/zero air and subtraction of the spectrum of unreacted HFPI.

We focus on Cl initiation to investigate products from HFPI because the OH experiments involve ozone, which blocks out parts of the IR spectrum and leads to a variety of secondary products that congest the spectrum. By contrast Cl_2_ is IR-inactive and leads to simpler spectra. A mixture of 0.064 torr HFPI and 0.68 torr Cl_2_ was made up to 1 bar with zero air. Spectra were recorded at 1 cm^−1^ resolution with 38 co-added scans. New peaks appeared during the 365 nm UV irradiation of this mixture (see Figure 6). Some of these can be assigned to hexafluoroacetone ((CF_3_)_2_CO), carbonyl difluoride (CF_2_O), and (CF_3_)_2_C(Cl)NO (tentatively identified, see below), and the lines at 2400–3100 cm^−1^ are from HCl.

**Figure 7:**
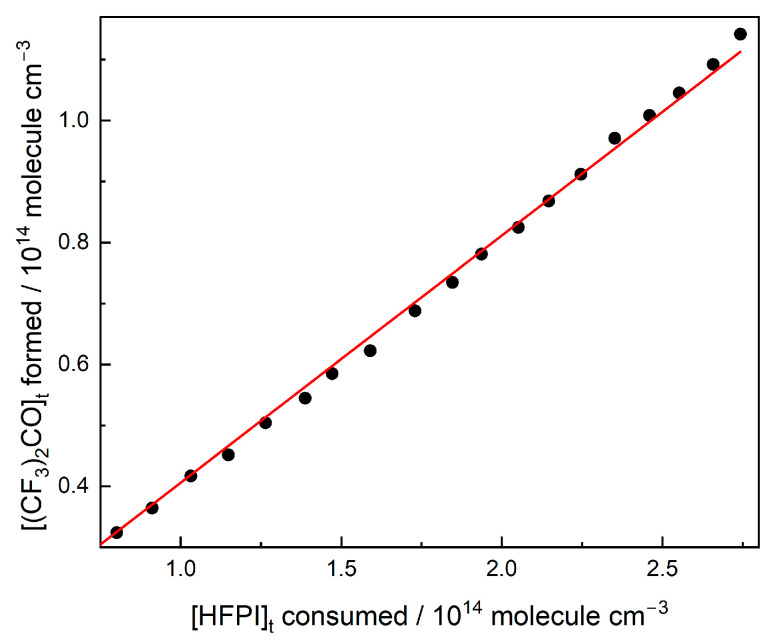
Formation of (CF_3_)_2_CO as a function of HFPI consumption.

Spectral subtractions were used to calculate the yield of (CF_3_)_2_CO in comparison to the consumption of HFPI, as shown in Figure 7. The slope for the plot of concentration of (CF_3_)_2_CO formed at different time intervals versus the concentration of HFPI consumed gave the yield of (CF_3_)_2_CO as (41 ± 5)%. For the yield of this species and the others, any consumption of HFPI by secondary radicals will contribute to the observed HFPI loss and product formation, so our quoted yields are effectively total quantities and may not refer to the Cl-atom chemistry alone.

The yield of carbonyl difluoride (CF_2_O) was determined in the same way, as (17 ± 5)% (see Supplementary Materials for a yield plot, Figure S1).

**Figure 8:**
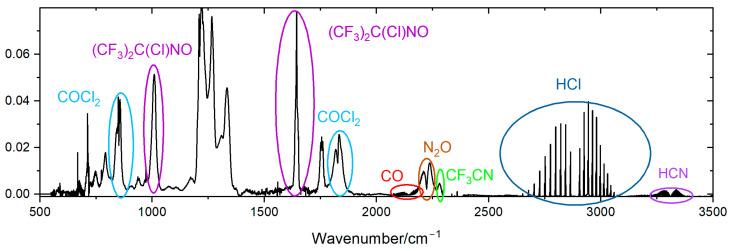
IR spectrum of products, after subtraction of CF_2_O, (CF_3_)_2_CO, and unreacted HFPI, highlighting peaks from HCl, HCN, N_2_O, CO, and COCl_2_ with tentative assignments for (CF_3_)_2_C(Cl)NO and CF_3_CN.

The residual spectrum obtained after subtracting (CF_3_)_2_CO, CF_2_O, and HFPI from the UV-irradiated products is shown in Figure 8 and features a strong 1644 cm^−1^ peak. Simple nitroso compounds exhibit a ca. 1500 cm^−1^ N-O stretching band that can be shifted to higher values by electronegative substituents, as seen in CF_2_ClNO, where NO stretching has a strong band at ~1600 cm^−1^ [17]. Based on this, and from mechanistic considerations (see Section 3.2), a candidate is (CF_3_)_2_C(Cl)NO. B2PLYP/6-31G(d) anharmonic calculations for this molecule yield 1641 cm^−1^, close to the observed band at 1644 cm^−1^. The first overtone is predicted to have significant intensity at 3255 cm^−1^, but is obscured by HCN (see below). (CF_3_)_2_C(Cl)NO may also account for the product peak observed at 1000 cm^−1^ (see Figure 8). Although C-Cl stretching is typically observed at 550–850 cm^−1^, in the molecule (CF_3_)_2_CCl_2_, strong C-Cl stretches occur at 912 and 942 cm^−1^ [18]. The presence of NO appears to cause a further shift to higher frequency, as seen in our anharmonic calculation for (CF_3_)_2_C(Cl)NO that yields C-Cl stretching at 1011 cm^−1^. Based on the approximate reproduction of the 1000 and 1644 cm^−1^ bands, we tentatively propose that (CF_3_)_2_C(Cl)NO is a product.

Figure 8 reveals the presence of other products. HCl is clearly observed but hard to quantify because of wall losses. There are spectra from phosgene (COCl_2_), nitrous oxide (N_2_O), and hydrogen cyanide (HCN). Comparison with reference spectra for these three species indicates yields of ca. 12%, 14%, and 18%, respectively (see Supplementary Material, Figures S2–S4). The weak signals imply high uncertainty, perhaps up to a factor of two, but qualitative identification is of interest. The first two species are artifacts from the long photolysis times and high concentrations of reactants, Cl and HFPI, needed for the experiments on this relatively unreactive system, because they involve products from at least two successive reactions of these reactants. In the atmosphere, at much greater dilutions, overall reactions that require successive encounters with two Cl radicals or the nitrogen atom contributions from two imine molecules will be negligibly slow. The shoulder on the N_2_O band at ~2280 cm^−1^ may reflect C-N stretching in trifluoroacetonitrile (CF_3_CN) [15]. It is too small to quantify reliably but appears to correspond to a CF_3_CN yield of the order of 1%. The HCN plot (Figure S2) is not linear through the origin, so the quoted yield is indicative only and the curvature suggests that multiple steps are needed to make HCN. It has been known for some time that photolysis of carbon monoxide (CO) mixed with chlorine makes COCl_2_ [19], and a faint CO spectrum is highlighted in Figure 8. A potential source of the CO is photolysis of (CF_3_)_2_CO [20]. Some of the peaks in the typical C-Cl range below 800 cm^−1^ might reflect species formed from radical plus Cl_2_ chemistry, but they cannot be identified from this alone.

## 3. Discussion

### 3.1. Comparison with Prior Kinetic Studies

There are no previous kinetic studies of (CF_3_)_2_CNH or indeed any R-N=H molecules we are aware of, but we can compare our measured kinetics to data for the isoelectronic molecule (CF_3_)_2_CCH_2_ [21]. The latter species is 15 times more reactive towards OH and 4 × 10^4^ times more reactive towards Cl atoms, suggesting perhaps that different reaction pathways are involved.

### 3.2. Mechanistic Interpretation

**Figure 9:**
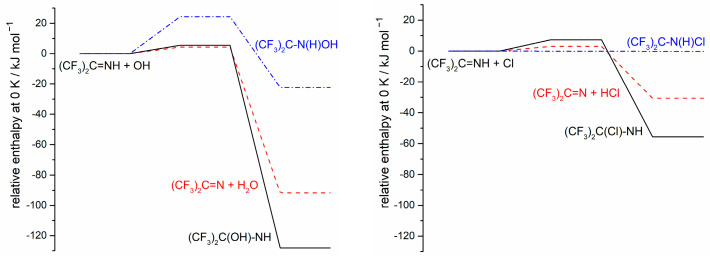
Relative reactant, TS, and product enthalpies for OH and Cl reactions with HFPI. Solid black line, addition to C; dot-dash blue line, addition to N; dashed red line, H abstraction.

We can suggest reaction paths that would account for many of the observed products. In principle, Cl atoms could add to either end of the C=N bond in HFPI or abstract the H atom from the N-H bond. Initial computations with density functional theory (DFT, see Section 4.3) are shown in Figure 9. Any pre- or post-transition state complexes are not included. 

The calculations indicate that Cl addition to N has ∆H ~ 0 and ∆S < 0; therefore, it is thermodynamically unfavorable. Cl atoms readily add to C=C bonds to form adducts that are stable at room temperature, and this difference may account for the much greater reactivity of (CF_3_)_2_CCH_2_ noted above. In HFPI, DFT indicates that addition to C has a barrier of ca. 7 kJ mol^−1^ and ΔH(0 K) = −56 kJ mol^−1^, and H-abstraction has a barrier of ca. 3 kJ mol^−1^ with ΔH(0 K) = −31 kJ mol^−1^. Given the typical accuracy of DFT, while we can rule out Cl addition to the N atom, it is not possible to distinguish between the other two pathways on the basis of the computed barriers. We also note that these barriers may be underestimated due to basis set superposition error. For OH + HFPI, the two most favorable channels are again addition to C and H-abstraction, and N addition is disfavored by a significant barrier. Thus, the products from attacks by either radical may be similar.

**Figure 10:**
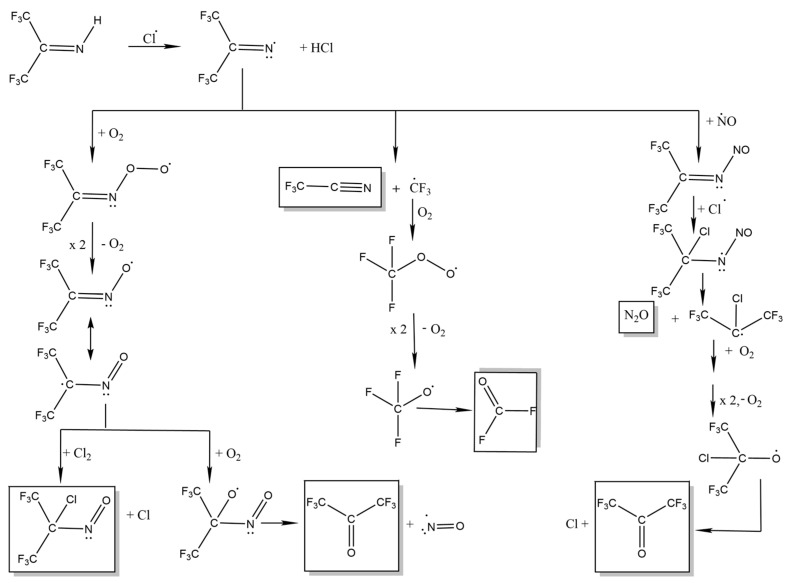
Possible mechanism for the reaction of Cl with HFPI initiated by H-atom abstraction.

The observed formation of HCl is consistent with H-abstraction and some possible subsequent steps are shown in Figure 10. These are based on a typical oxidation path for radicals: R• + O_2_ → RO_2_; self-reaction of two of these peroxy radicals to yield two alkoxy RO• species + O_2_; and then, RO• → R′=O + R″• to create carbonyl species. This mechanism would account for some of the products detected, although not COCl_2_ and HCN. We also note that Sulbaek Andersen et al. [15] measured a Cl + CF_3_CN rate constant that is three times larger than we find for Cl + HFPI, so that only a small, steady state [CF_3_CN] might be expected in our experiment. We speculate that Cl-initiated processing of CF_3_CN might lead to CN radicals. If so, these could rapidly abstract H from HFPI, leading to the observed HCN product.

**Figure 11:**
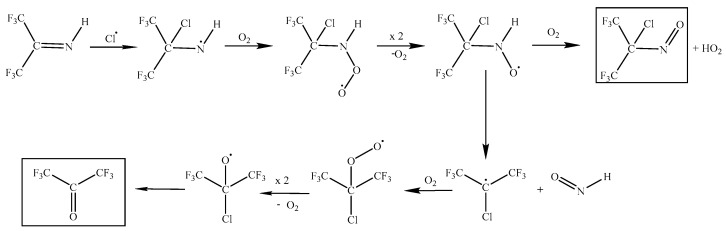
Possible mechanism for the reaction of Cl with HFPI initiated by addition to the C atom.

A second possible mechanism, starting with addition of Cl to the π bonded C atom in HFPI, is shown in Figure 11. It leads to two of the major observed products, (CF_3_)_2_CO and (CF_3_)_2_C(Cl)NO. The predicted HNO by-product would rapidly react with Cl atoms to make HCl + NO. The addition and abstraction pathways both yield (CF_3_)_2_CO, with only abstraction yielding CF_2_O. The observed (CF_3_)_2_CO: CF_2_O ratio of ca. 2.4:1 is consistent with both mechanisms operating in parallel. 

Again, we highlight that the laboratory conditions are not equivalent to the atmosphere, because [HFPI] and [Cl_2_] are necessarily much higher. If the mechanisms in Figure 10 and Figure 11 are correct, then in the atmosphere we may expect the main degradation products of HFPI to be (CF_3_)_2_CO, NO, CF_3_CN, and CF_2_O.

### 3.3. Atmospheric Lifetime of HFPI and Its Global Warming Potential

Given that the rate constant for HFPI removal by Cl is about 50 times smaller than that for removal by OH, and that Cl is about 30 to 1000 times less abundant in the atmosphere than OH [22,23], the Cl reaction makes a negligible contribution to atmospheric loss.

The lifetime τ_OH_ of HFPI with respect to removal through reaction with hydroxyl is estimated as

τ_OH_^−1^ = *k*_HFPI + OH_ [OH] (1)

and with the globally averaged OH in the troposphere of ca. 10^6^ molecule cm^−3^ we obtain τ_OH_ = 0.75 years.

The methods described by Hodnebrog et al. [2] are then applied to derive the GWP. A lifetime-based correction factor of 0.69 to the radiative efficiency accounts for mixing effects in the atmosphere and yields an effective R.E. of 0.23 W m^−2^ ppb^−1^. This leads to the GWP values listed in Table 4. These have been calculated for several time horizons. The most typically considered is the 100-year time frame.

The value of GWP_100_ = 64 is comparable to those for saturated hydrofluorocarbon molecules. This is primarily because of the modest reactivity of this imine, caused perhaps by the two electron-withdrawing CF_3_ groups inhibiting radical attack at the C=NH moiety.

If other atmospheric removal mechanisms operate, the GWP would be lower. Three are discussed here.

(i) With an upper limit to ozone in the atmosphere of 100 ppb in highly polluted areas, the small rate constant determined in Section 2.2 indicates that consumption of imine by atmospheric ozone, a pathway for some unsaturated VOCs, is entirely negligible for HFPI.
(ii) A second possibility is UV photolysis. The UV absorption spectrum by Toby et al. shows a broad peak centered near 250 nm, which has minor intensity at 300–320 nm [24]. An initial assessment of excited electronic states of HFPI via time-dependent density functional theory confirms that its longest wavelength UV transition is centered at approximately 245 nm, with no unobserved longer wavelength peaks. Thus, the main UV absorption is outside the actinic region of ground-level sunlight, ca. 290–400 nm, and the derived photolysis rate in overhead sunlight is ~10^−6^ s^−1^, if every photon absorbed led to dissociation. This implies a lifetime of at least 24 days. The measurements by Toby et al. [24] also showed that, at tropospheric pressures, the quantum yield for dissociation is small, ca. 10^−3^ or less, so the lifetime with respect to photolysis becomes at least 60 years. Therefore, this process has a negligible influence on the overall atmospheric lifetime of HFPI.
(iii) A third potential removal pathway is via absorption into cloud water droplets and rainout. There is little information available about the water solubility of HFPI. We note that a synthesis of HFPI involves the addition of ammonia to hexafluoro acetone to make a (CF_3_)_2_C(OH)NH_2_ intermediate, followed by dehydration with phosphorous oxychloride to yield HFPI [25]. If this route is readily reversible, then a typical average lifetime for soluble species removed by cloud droplets is transport-controlled and of the order of a week [26]. Shortening the overall atmospheric lifetime by around a factor of 40 in this way would have a similar impact on the GWP, i.e., leading to a GWP_100_ of around two. Further experiments are needed to evaluate the solubility of HFPI and its possible hydrolysis.

## 4. Materials and Methods

### 4.1. Materials

The HFPI was obtained from Synquest Laboratories (Alachua, FL, USA, 99%). Other reagents used were CH_3_F (Synquest, 99%), CH_3_CF_2_H, CF_3_CFH_2_ (commercial canned “air” dusters), Cl_2_ (Matheson, Irving, TX, USA, 99.5%), H_2_ (MG Industries, Vacaville, CA, USA, 99.999%), Ar (Airgas, Radnor, PA, USA, UHP 99.9999%), O_2_ (Airgas, UHP 99.999%), and zero air (Airgas, Industrial grade). A liquid nitrogen trap was used to separate the HFPI initially, and the other organic species and Cl_2_, followed by freeze–pump–thaw cycles at 77 K. Ozone was synthesized by passing pure O_2_ through an ozone generator (A2Z Ozone, Louisville, KY, USA) and the O_3_ was separated from unreacted O_2_ in a trap filled with a silica gel and cooled with an acetone/dry ice slush bath. UHP Ar, N_2_, O_2_, and H_2_ were used directly from their cylinders.

### 4.2. Experimental Methods

We have previously provided details of the apparatus [27]. A grease-free Pyrex gas-handling line was used to store reagent gases, to purify them via low-temperature distillations, and to prepare known mixtures with partial pressures measured with capacitance manometers. Mixtures were made up to 1 bar pressure with a bath gas. For IR measurements on HFPI and kinetic studies, this bath gas was argon. For product studies, to simulate atmospheric conditions, the bath gas was “zero air”, an O_2_/N_2_ blend which contains no CO_2_ to interfere with IR measurements. Gas samples were loaded into a 100 cm^3^ quartz multipass IR cell with a path length of 2.4 m, mounted in a Nicolet iS50 Fourier-Transform infrared spectrometer (Thermo Scientific, Waltham, MA, USA). HFPI and other species were quantified by their IR spectra. A 1 cm^−1^ resolution was employed and 38 scans were co-added over 1 min to improve the signal-to-noise ratio. All experiments were conducted at the laboratory room temperature.

The IR cell was irradiated with UV light to initiate chemical reactions by continuous photolysis of precursor molecules, ozone, or molecular chlorine, which generated reactive radicals. Two mercury lamps were used. One was an Upland UVGL-25 Compact lamp (UVP, Upland, CA, USA) for 254 nm photolysis of O_3_ to produce O(^1^D), which reacted with excess H_2_ as a source of OH. The other was a Pen-Ray 11SC-2.12 lamp(Analytik Jena, Upland, CA, USA), with an added Pyrex filter, for 365 nm radiation to generate atomic Cl directly from Cl_2_. Reactivity was quantified by the relative rate method [28]. When the same species simultaneously consumes HFPI and an added reference compound, with rate constants *k*_HFPI_ and *k*_ref_, respectively, one can write

(2) $$ln\left(\frac{{\left[HFPI\right]}_{0}}{{\left[HFPI\right]}_{t}}\right)=\frac{k_{HFPI}}{k_{ref}}ln\left(\frac{{\left[ref\right]}_{0}}{{\left[ref\right]}_{t}}\right)$$

The concentration ratios are derived from the changes in the IR absorbances. Provided *k*_ref_ is already known; *k*_HFPI_ is obtained via the slope of a plot of the logarithms of the reactant and reference concentration ratios. Figure 4 is an example. Data were initially processed in Excel (Office 365, Microsoft, Redmond, WA, USA) and graphs and linear fits were made with OriginPro 10.1 (OriginLab Corp., Northampton, MA, USA).

Product yields are expressed as the amount of a given species formed, ratioed to the amount of reactant consumed. This requires absolute IR cross sections or integrated band strengths that are sometimes available in the literature, and, in some cases, are synthesized to create reference spectra with quantified cross sections. These cross sections σ in cm^2^ molecule^−1^ are defined via

A ln 10 = σ c ℓ (3)

where A is the measured base 10 absorbance at a given wavenumber, c is the concentration of species in molecule cm^−3^, and ℓ is the path length, 240 cm. c is obtained from the partial pressure and temperature via the ideal gas law. The factor of ln 10 puts σ on the customary base e scale. Changes in reactant absorbance during the course of a reaction were derived either from a least-square fit of the ratio of the spectrum (A vs. wavenumber *v*) to the initial spectrum (“spectral subtractions”). Products were quantified via spectral subtractions or with integrated band strengths, based on the integration of known cross sections over a selected band, with reference to our library of measured spectra or to spectra downloaded in 2016 from the Pacific Northwest National Laboratory infrared database [16].

**Figure 12:**
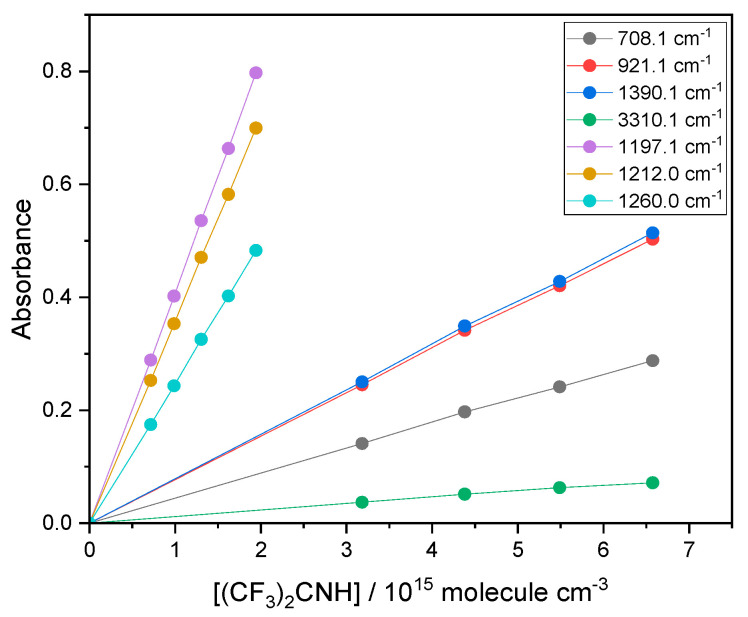
Beer–Lambert plots for HFPI at 292 ± 2 K in 1 bar (750 torr) Ar.

Equation (3) embodies the Beer–Lambert Law, which was verified by linear plots of A vs. c for several strong features of the spectrum of HFPI, by varying the concentration and checking for a proportional absorbance (no saturation at band peaks), with partial pressures from 0.10 to 0.20 torr (1 torr = 1.33 mbar). This is demonstrated in Figure 12, where the linear fits had intercepts not significantly different from zero. Particularly strong peaks were observed near 1200 cm^−1^ and, to ensure Beer–Lambert behavior, smaller pressures of 0.006 to 0.022 torr were employed for this region. Proportionality between A and c was assumed moving forward.

### 4.3. Computational Methods

IR assignments for unknown molecules were assisted by theoretical modeling. Computations at the B2PLYP/6-31G(d) level of density functional theory [29] were carried out with the Gaussian16 program [30]. Results were combined with variational second-order perturbation theory (VPT2) [31,32] to generate predicted spectra, corrected for vibrational anharmonicity. The thermochemistry and barrier heights for initial attack of OH and Cl radicals on HFPI were estimated with M06-2X/6-311+G(2df,2p) density functional theory [33] with unscaled zero-point energy.

## 5. Conclusions

The first kinetic measurements for radicals with a R-N=H species indicate that 1,1,1,3,3,3-hexafluoroprop-2-imine is moderately reactive towards OH and reacts slowly with Cl. The results allow an initial evaluation of the global warming potential. Studies of the products of Cl-initiated oxidation provide some insight into likely degradation products in the atmosphere, and, along with density functional theory estimates for barriers and thermochemistry, suggest that the initial attack by radicals is through abstraction of the H atom and addition to the π-bonded C atom.

## Figures and Tables

**Table 1:** Initial partial pressures and relative rate constant ratio for OH + (CF_3_)_2_CNH with CH_3_F reference.

p(HFPI)/torr	p(CH_3_F)/torr	p(O_3_)/torr	p(H_2_)/torr	*k*_HFPI_/*k*_CH3F_
0.17	0.37	3.67	20.00	2.03 ± 0.06
0.14	0.32	3.17	17.29	2.33 ± 0.08
0.15	0.33	3.29	17.90	2.14 ± 0.08

**Table 2:** Initial partial pressures and relative rate constant ratio for OH + (CF_3_)_2_CNH with CH_3_CHF_2_ reference.

p(HFPI)/torr	p(CF_2_HCH_3_)/torr	p(O_3_)/torr	p(H_2_)/torr	*k*_HFPI_/*k*_CF2HCH3_
0.15	0.33	2.40	19.01	1.33 ± 0.02
0.22	0.47	2.91	22.27	1.34 ± 0.02
0.18	0.39	2.40	18.41	1.32 ± 0.04
0.15	0.31	1.94	14.82	1.39 ± 0.04

**Table 3:** Initial partial pressures and relative rate constant ratio for Cl + HFPI with CF_3_CH_2_F reference.

p(HFPI)/torr	p(CF_3_CH_2_F)/torr	p(Cl_2_)/torr	*k*_HFPI_/*k*_CF3CH2F_
0.097	0.099	3.218	0.57 ± 0.01
0.059	0.063	2.045	0.57 ± 0.01
0.083	0.089	2.860	0.57 ± 0.01

**Table 4:** Global warming potentials for (CF_3_)_2_CNH based on removal by OH.

Time Horizon, Years	GWP ^a^
20	234
50	110
100	64
500	18

^a^ Uncertainty ± 30%.

## Data Availability

The original contributions presented in this study are included in the article. Further inquiries can be directed to the corresponding author.

## Supplementary Materials

The following supporting information can be downloaded at: https://www.mdpi.com/article/10.3390/molecules31030424/s1, Figure S1: Formation of CF_2_O as a function of HFPI loss; Figure S2: Formation of HCN as a function of HFPI loss; Figure S3: Formation of N_2_O as a function of HFPI loss; Figure S4: Formation of COCl_2_ as a function of HFPI loss; Table S1: IR cross sections of (CF_3_)_2_CNH; Table S2: Cartesian coordinates of reactants, transition states and products for OH and Cl reactions with HFPI (see Figure 9), in units of 10^−10^ m, derived with M06-2X/6-311+G(2df,2p) density functional theory.
