# Supplementary material for: Measurements of Radical Reactivity with an Imine, (CF3)2CNH: Rate Constants for Chlorine Atoms and Hydroxyl Radicals and the Global Warming Potential
Source: Molecules. 2026 Jan 26;31(3):424. doi: 10.3390/molecules31030424 (PMC12899651; doi:10.3390/molecules31030424)
Supplement: Supplementary file 1 [file molecules-31-00424-s001.zip › Supplementary figures and table.pdf]

Supplementary Information

Measurements of Radical Reactivity with an Imine, $(CF_3)_2CNH$: Rate Constants for Chlorine Atoms and Hydroxyl Radicals and the Global Warming Potential

Savi Savi and Paul Marshall *

Department of Chemistry and Center for Advanced Scientific Computing and Modeling, University of North Texas, 1155 Union Circle #305070, Denton, TX 76203, USA; savisavi@my.unt.edu
* Correspondence: paul.marshall@unt.edu

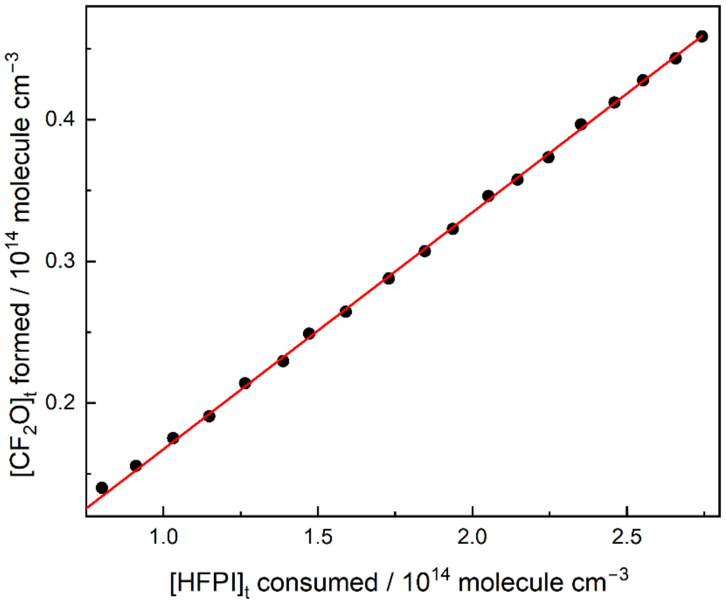

Figure S1. Formation of CF_2O as a function of HFPI loss.

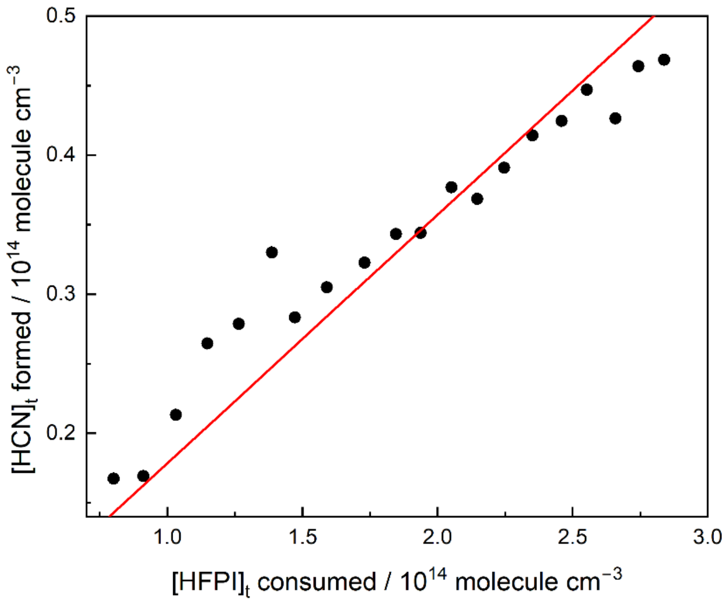

Figure S2. Formation of HCN as a function of HFPI loss.

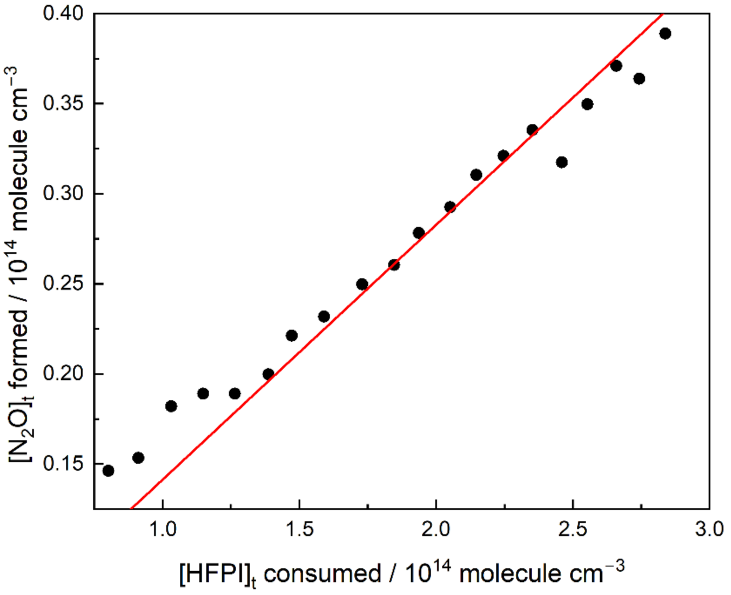

Figure S3. Formation of N_2O as a function of HFPI loss.

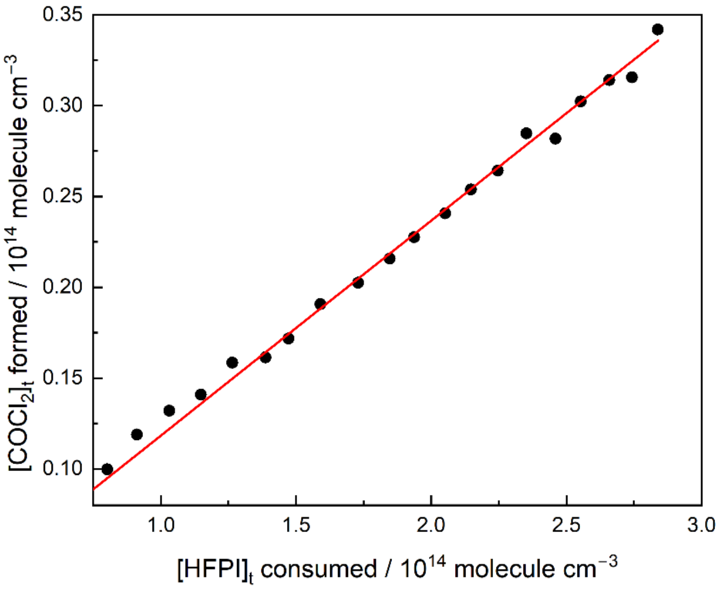

Figure S4. Formation of $COCl_2$ as a function of HFPI loss.

Table S2. Cartesian coordinates of reactants, transition states and products for OH and Cl reactions with HFPI (see Fig. 9), in units of 10^{-10} m, derived with M06-2X/6-311+G(2df,2p) density functional theory.

IMINE - (CF3)2CNH

C	-1.27834900	-0.12083300	-0.00026100
C	0.00066200	0.71495000	0.01334800
C	1.30180400	-0.08211700	0.00639200
N	0.03944200	1.96323400	0.01213700
H	-0.89640500	2.37081200	-0.01836100
F	-2.32202600	0.64142200	-0.31545100
F	-1.50727500	-0.65377700	1.19538300
F	-1.19258300	-1.10522000	-0.88709400
F	1.18805400	-1.15990200	0.78266100
F	2.31570500	0.64295600	0.43484500
F	1.57097000	-0.49719500	-1.23073000

TS for abstraction of H by Cl atom

C	-0.30553100	1.07079800	-0.00001400
C	0.24047400	-0.37632500	-0.00005300
C	1.77300300	-0.53680100	0.00001800
N	-0.45045100	-1.40548900	-0.00016600
H	-1.62777700	-1.32570000	-0.00018300
F	-1.03905400	1.27524200	1.07835300
F	-1.03881300	1.27535200	-1.07853000
F	0.69663900	1.93924200	0.00014600
F	2.27980900	0.04393800	-1.07920900
F	2.11422200	-1.80842600	-0.00005100
F	2.27969500	0.04378800	1.07937900
Cl	-3.12348300	-0.86494800	0.00005000

Product from abstraction of H by Cl atom

C	1.30426700	-0.07229600	0.00241000
C	0.00000100	0.73464300	-0.00000700
C	-1.30426600	-0.07229900	-0.00241300
N	-0.00000300	1.97124800	-0.00000600
F	2.32744300	0.69025400	0.33868300
F	1.52583900	-0.57553700	-1.20496700
F	1.21279500	-1.07799600	0.86430400
F	-1.21275000	-1.07808800	-0.86419700
F	-2.32742500	0.69021900	-0.33881800
F	-1.52590300	-0.57541100	1.20500600

HCl

Cl	0.00000000	0.00000000	0.07094200
H	0.00000000	0.00000000	-1.20601700

TS for addition of Cl atom to doubly bonded carbon

C	-1.28357100	0.51753100	0.06980400
C	0.00631600	-0.04615000	-0.55549300
C	1.32516500	0.45841800	0.04597800
N	0.05353800	-0.52477400	-1.74417300
H	-0.88505400	-0.71114100	-2.10439500
F	-2.33986600	0.00891000	-0.55532300
F	-1.28502500	1.83704000	-0.11544500
F	-1.40125000	0.28274000	1.36106900
F	1.53485000	1.69722000	-0.40002000
F	2.33766800	-0.29724300	-0.32471400
F	1.27598100	0.50341500	1.36990000
Cl	-0.05167000	-2.20488000	0.29015600

Product for addition of Cl atom to doubly bonded carbon

C	1.27029800	-0.42260400	0.09987200
C	-0.00274500	0.30624800	-0.40181200
C	-1.29732300	-0.39630400	0.06402500
N	-0.01747800	0.38034200	-1.82237800
H	0.85064600	0.84010200	-2.11149400
F	2.34655600	0.21866900	-0.35067300
F	1.30502000	-1.66025600	-0.37695100
F	1.33433300	-0.47400600	1.41765100
F	-1.35824800	-1.61895900	-0.44665100
F	-2.35305000	0.28387400	-0.35304500
F	-1.35189000	-0.49176500	1.38615900
Cl	0.00857900	1.95620300	0.28277800

TS for addition of Cl atom to nitrogen

C	-1.47252200	-0.61480400	0.02734800
C	-0.07834600	-0.16330900	-0.32832900
C	0.29553800	1.26865900	-0.05239500
N	0.83358400	-0.99873800	-0.74937500
H	0.51549200	-1.95509200	-0.87437500
F	-1.54954200	-1.94672200	-0.03034100
F	-2.38063300	-0.11925600	-0.80726900
F	-1.80558600	-0.23288600	1.25837200
F	-0.76573000	2.05872600	-0.20582800
F	1.25388400	1.67689800	-0.87118300
F	0.73106300	1.41977900	1.19897400
Cl	2.46060700	-1.15916900	0.19739600

Product of addition of Cl atom to nitrogen

C	-1.49523400	-0.55087400	0.02464300
C	-0.07634800	-0.17481500	-0.27070000
C	0.36412000	1.23652900	-0.05167900
N	0.81816000	-1.10786500	-0.63815100
H	0.44629400	-2.04593300	-0.72577300
F	-1.63219800	-1.88276600	-0.01067100
F	-2.35233300	-0.03842600	-0.85646300
F	-1.87831500	-0.13254600	1.23218700
F	-0.66841400	2.06736000	-0.18361500
F	1.30680300	1.58612100	-0.92256200
F	0.86773300	1.42353900	1.17393000
Cl	2.36952300	-1.20432900	0.18141100

OH radical

O	0.00000000	0.00000000	0.10788000
H	0.00000000	0.00000000	-0.86303700

TS for addition of OH radical to nitrogen

C	1.43280000	0.08431800	0.02191900
C	-0.02658700	0.34647100	-0.29741800
C	-1.00190000	-0.78411100	-0.04108200
N	-0.46885500	1.46858400	-0.70098400
H	0.23140700	2.20061700	-0.78990400
F	2.11204500	1.22956900	0.03728100
F	1.98869800	-0.70795200	-0.89009700
F	1.56601600	-0.50052300	1.20733900
F	-0.47201000	-1.94626700	-0.42550900
F	-2.13217000	-0.59684300	-0.70457900
F	-1.28523500	-0.87821900	1.25369600
O	-1.62372700	2.24444600	0.43242600
H	-2.38157000	2.28576700	-0.16631700

Product of addition of OH radical to nitrogen

C	1.44613600	0.03143800	0.03053900
C	-0.00235400	0.36483600	0.09824000
C	-1.03414200	-0.70808400	0.00512400
N	-0.37079700	1.64019800	-0.22211800
H	0.31564800	2.36037100	-0.03524800
F	2.17411100	1.14492900	0.21182300
F	1.81696100	-0.48432000	-1.14810400
F	1.79990500	-0.84634300	0.96519400
F	-0.46302800	-1.89594600	-0.18308400
F	-1.87472400	-0.48779500	-1.01964000
F	-1.78914900	-0.79556000	1.10167100
O	-1.62220100	2.02933700	0.24691400
H	-2.17698300	2.07971200	-0.53940500

TS for addition of OH radical to doubly bonded carbon

C	1.30190300	-0.26473200	0.02415800
C	-0.00194000	0.39484900	-0.45108200
C	-1.29657800	-0.28212100	0.00238300
N	-0.07217400	1.17870000	-1.45492400
H	0.85771500	1.47843100	-1.75107200
F	2.34230100	0.35988700	-0.51959900
F	1.30543600	-1.52882900	-0.40374700
F	1.45614200	-0.27392900	1.33257400
F	-1.58727500	-1.27713800	-0.83228800
F	-2.30638400	0.57703500	-0.01205700
F	-1.18574500	-0.78829900	1.22233800
O	0.01755200	1.89310600	0.85806400
H	-0.73349300	2.41930200	0.53329000

Product of addition of OH radical to doubly bonded carbon

C	1.27714900	-0.24327400	0.01508700
C	0.00055800	0.62917000	-0.05261200
C	-1.27707700	-0.23676600	-0.01688500
N	-0.01263200	1.37728600	-1.27960200
H	0.76924900	2.03536800	-1.20825700
F	2.34362900	0.53367000	-0.16723300
F	1.26549900	-1.16356500	-0.94092200
F	1.39955200	-0.84486000	1.18406600
F	-1.44074800	-0.93107500	-1.12813800
F	-2.32623100	0.58145400	0.12771800
F	-1.27335600	-1.07492700	1.01143300
O	0.04531800	1.44426300	1.07407000
H	-0.76225200	1.96847600	1.11706000

TS for abstraction of H by OH radical

C	0.79384200	-0.81275400	-0.00606000
C	-0.03914100	0.47791600	0.02435200
C	-1.56011400	0.29486800	0.00184500
N	0.42883900	1.62996700	0.04478300
H	1.52398600	1.73571700	0.02953300
F	1.73161400	-0.77732200	0.94481600
F	1.39894200	-0.94542800	-1.17607400
F	0.04416700	-1.88380100	0.20691000
F	-1.90537500	-0.46165200	-1.03838200
F	-2.17965000	1.45276700	-0.09411700
F	-1.96703100	-0.31317300	1.11358200
O	2.88255400	1.56873800	-0.08098000
H	3.14218700	0.90192100	0.57337700

H2O

O	0.00000000	0.00000000	0.11637300
H	0.00000000	0.76143800	-0.46549000
H	0.00000000	-0.76143800	-0.46549000
